# Assessment of predictors for difficult intubation and laryngoscopy in adult elective surgical patients at Tikur Anbessa Specialized Hospital, Ethiopia: A cross-sectional study

**DOI:** 10.1016/j.amsu.2022.103682

**Published:** 2022-04-28

**Authors:** Tamirat Alemayehu, Mulualem Sitot, Abebayehu Zemedkun, Siryet Tesfaye, Dugo Angasa, Fasil Abebe

**Affiliations:** a Tikur Anbessa Specialized Hospital, Addis Ababa, Ethiopia; b Addis Ababa University, College of Health Sciences, Department of Anesthesia, Addis Ababa, Ethiopia; c Dilla University, College of Health Sciences and Medicine, Department of Anesthesiology, Dilla, Ethiopia; d Hawassa University, Colleges of Medicine and Health Sciences, Hawassa, Ethiopia; e Ambo University, Colleges of Medicine and Health Sciences, Ambo, Ethiopia

**Keywords:** Difficult airway, Difficult laryngoscopy, Difficult intubation, Intubation, Predictors

## Abstract

**Background:**

General anesthesia is not without morbidity. One of the well-known life-threatening events associated with general anesthesia is difficult airway which can happen during induction of anesthesia while attempting to insert the endotracheal tube with the aid of a laryngoscope. Difficult intubation, inadequate ventilation, and esophageal intubation are the principal causes of death or brain damage related to airway manipulation.

**Objective:**

The main objective of this study was to assess the magnitude and predictors for difficult laryngoscopy and intubation among surgical patients who underwent elective surgery under general anesthesia with endotracheal intubation at Tikur Anbessa Specialized Hospital from February 1 to March 30, 2019.

**Materials &method:**

An institutional based cross sectional study was conducted from February 1 to March 30, 2019 on patients who underwent elective surgery under general anesthesia with endotracheal intubation. Data on socio-demographic characteristics, preanesthetic airway assessment and laryngoscopic view were collected. Data were analyzed by SPSS Version 20.0. Chi- square test, binary logistic regression and multivariate analysis were performed. Tables and texts were used to present data. A p value less than 0.05 was considered as statistically significant.

**Results:**

The magnitude of difficult laryngoscopy, difficult intubation, and failed intubation were 12.2%, 6.1%, and 0.67%, respectively. Upper Lip Bite Test (ULBT) had a higher sensitivity (90.2%) and negative predictive value of 85.3%. Mallampati had a sensitivity of 45.8% and negative predictive value of 86% in predicting difficult laryngoscopy. Mallampati grade, thyromental distance and ratio of height to thyromental distance (HRTMD) have also showed greater sensitivity (69.6%, 58.3% and 47.8%, respectively) when compared to other tests in predicting difficult intubation. Mallampati class, upper lip bite test (ULBT) and inter-incisor distance (IID) are independent predictors for difficult laryngoscopy (p < 0.05). Furthermore, Mallampati class, Thyromental distance and ratio of height to thyromental distance (HRTMD) are identified as independent predictors of difficult intubation (p < 0.001).

**Conclusion:**

and recommendation: Mallampati class, Thyromental distance and Ratio of height to Thyromental distance (HRTMD**)** can predict the probability of difficult endotracheal intubation in adult patients. Whereas, Mallampati class and upper lip bite test (ULBT) predicts higher probability for difficult laryngoscopy.

## Highlights

• The magnitude of difficult laryngoscopy and intubation were 12.2% and 6.1%, respectively.
• Mallampati class, TMD and HRTMD were predictors of difficult intubation.
• Upper Lip Bite Test had a higher sensitivity to predict difficult laryngoscopy.
• Mallampati class, Upper Lip Bite Test and IID predicts difficult laryngoscopy.

## 1 Background

The first major responsibility for the anesthesia professional is to provide adequate ventilation and oxygenation by securing the patient's airway. Preoperative assessment of the patient's airway facilitates the anesthetists to predict the ease of visualizing the glottis and to perform intubation easily. Furthermore, Management of the difficult airway is one of the most relevant issues and core competency for practicing anesthetists [[1], [2]]. Maintaining a patent airway is must for adequate oxygenation and ventilation and failure to do so, even for a brief period of time can be life threatening. Difficult airway management can result in patient harm from relatively minor problems such as oral trauma up to an increased risk of aspiration, hypoxia, cerebral damage and death from inability to oxygenate [3]. Appropriate management of the difficult airway constitutes an important place in the prevention of mortality and morbidity associated with anesthesia. Failure to assess for and identify potential difficulty, or the application of poor judgment in management planning, may contribute to a poor outcome [[4], [5]].

The term “difficult airway” covers a spectrum ranging from problems in ventilating a patient'slung with a face mask or supraglottic airway to problems in intubating and extubating a patient's trachea. A recent guideline update defines the difficult airway as an airway for which an experienced practitioner anticipates or encounters difficulty with facemask ventilation, tracheal intubation, or supraglottic airway use or recognizes the need for an emergency surgical airway [2,6]. The prevalence of difficult laryngoscopy (inability to visualize any portion of the vocal cords after multiple attempt at laryngoscopy) has been reported to range between 5% and 20%, and a variety of physical examination tests have been used to estimate its presence [2,4,[6], [7], [8]]**.** Difficult endotracheal intubation is defined as endotracheal intubation requiring multiple attempts [7,14]. The incidence of failed intubation is approximately 1 in 1000 and the incidence of cannot intubate cannot ventilate is approximately 1 in 2800–20,000 [9,10].

Among the strategies proposed to decrease morbidity and mortality related to difficult tracheal intubation (DTI), the role of its predictors remains a matter of debate [9]. Several clinical signs have been identified as predictors of difficult laryngoscopy or difficult tracheal intubation (DTI). These include the Mallampati score, the Thyromental Distance (TMD), Upper Lip Bite Test (ULBT), Sterno-Mental Distance (SMD), Ratio of height to Thyromental distance (HRTMD), and Inter Incisor Distance (IID). However, the sensitivity, specificity, positive and negative predictive values of these signs is a matter of debate and it requires set-up based investigation [2, 7, 8, [11], [12], [13], [14], [15], 25]. The main objective of this study was to determine magnitude and predictors for difficult intubation and laryngoscopy in adult elective Surgical Patients who underwent surgery at Tikur Anbessa Specialized Hospital Ethiopia.

## 2 Method and materials

The study was done after obtaining a letter for approval by institutional ethics committee of Addis Ababa University to conduct this study. An institutional based cross-sectional study was conducted at Tikur Anbessa Specialized Hospital from February 1 to March 30, 2020. All adult patients who underwent elective surgery under general anesthesia with endotracheal intubation and full-filled the inclusion criteria were included. Patients with an anticipated difficult airway, emergency and pediatric patients were excluded from the study. A pretested and structured questionnaire was prepared to collect data from the patients. Independent variables like airway parameters were collected by observing and measuring each airway assessment test, and the data for dependent variable was collected during the induction phase of anesthesia by qualified duty free anesthetist by observation. Data regarding the grade of laryngoscopy were collected from anaesthetic record sheet. Two qualified anesthetists were selected for data collection based on the capability of being free during the data collection period and experience of data collection. Half-day training was given for qualified anesthetists, who were involved in the data collection process. Furthermore, Informed consent was taken from each patient orally before data collection. This study was registered at www.researchregistry.com with Research Registry UIN: researchregistry 6882. This work also reported in line with STROCSS 2021 criteria [16].

### 2.1 Sample size determination and sampling technique

The sample size was calculated based on a previous research done in Gondar University, North Ethiopia [25] which showed magnitude of difficult intubation was 9% (p = 0.09) and by assuming a confidence level of 95% and margin of error 0.05, the required sample size was 148 patients by considering non response rate of 15%.

Systematic random sampling technique was employed to select study participants on daily operation schedule. From situational analysis 308 patients were operated on elective schedule for 2 month. The sampling interval; K was determined using: K=N/n; 308/148 = 2.

Therefore, the sampling interval was two and the first study participant (random start) was selected using lottery method from the daily operation schedule list. Then, every second cases were included in the study during the study period until the required sample size is reached.

### 2.2 Data analysis and interpretation

The data was cross-checked for completeness and consistency. Then it was entered on SPSS version 20 for analysis. Sensitivity, specificity, and positive and negative predictive value were calculated to assess the association between the outcome and exposure variables. Binary and multivariate logistic regression was used to assess the inﬂuence of each risk factors or airway parameters on the incidence of difficult laryngoscopy and difficult intubation. A P-value of less than 0.05 was considered as statistically significant.

### 2.3 Operational definition

**Mallampati grading: Grade I**: Visualization of the soft palate, fauces, uvula, anterior and the posterior pillars; **Grade II**: Visualization of the soft palate, fauces and uvula, **Grade III**: Visualization of soft palate and base of uvula; **Grade IV**: Only hard palate is visible, Soft palate is not visible at all.

**Thyromental Distance:** It is defined as the distance from the mentum to the thyroid notch while the patient's neck is fully extended.

### 2.4 Cormack and Lehane's laryngoscopic grade

Grade I – Visualization of entire laryngeal aperture.

Grade II – Visualization of only posterior commissure of laryngeal aperture.

Grade III – Visualization of only epiglottis.

Grade IV – Visualization of just the soft palate.

**Inter-Incisor Distance:** It is the distance between the upper and lower incisors. **Class 1:** lower incisors can bite the upper lip above the vermilion line; **Class 2:** lower incisors can bite the upper lip below the vermilion line; **Class 3:** lower incisors cannot bite the upper lip.

**Sterno-mental distance:** the distance from the suprasternal notch to the mentum and measured with the head fully extended on the neck with the mouth closed.

**Difficult laryngoscopy:** Cormack and Lehane grade III (epiglottis only) or grade IV view (soft palate only)

**Difficult intubation:** If a trained anesthetist using direct laryngoscopy takes more than 3 attempts or more than 10 min to complete tracheal intubation.

## 3 Result

### 3.1 Socio demographic data

One hundred forty eight (148) patients were included in the study. Majority of the participants 135 (91.2%) were within the age of 18–65 years. The body mass index data shows 121(81.8%) of the participants were above twenty five. The result also showed that out of 148 patients who underwent general anesthesia, 50(33.8%) were general surgery procedures, 18(12.2%) thoracic surgeries, 10(6.8%) orthopedic procedures, ENT 12(8.1%), 22 (14.9%) urologic and 18 (12.2%) gynecologic surgeries (Table 1)

**Table 1:** Socio demographic characteristics of participants who underwent elective surgery under general anesthesia with endotracheal intubation in Tikur Anbessa Specialized Hospital, Ethiopia.

Variables	Category	Frequency	Percentage
Age	18–65	135	91.2%
	≥65	13	8.8%
Sex	Male	82	55.4%
	Female	66	44.6%
BMI	≤25	121	81.8%
	>25	27	18.2%
Types of surgery	General surgery	50	33.8%
	ENT	12	8.1%
	Thoracic surgery	18	12.2%
	Orthopedic surgery	10	6.8%
	Neurologic surgery	18	12.2%
	Urologic surgery	22	14.9%
	Gynecologic surgery	18	12.2%

### 3.2 Magnitude and predictors of difficult laryngoscopy and intubation

In this study, we found that the magnitude of difficult laryngoscopy and intubation was 18/148 (12.2%) and 9/148 (6.1%), respectively. There was one case with failed intubation (0.67%). 16(10.8%) patients had TMD <6.5 cm of whom 12 had easy and 4 had difficult intubation (p < 0.05), whereas the rest 132(89.2%) had TMD >6.5 cm and only 5 of them were difficult to intubate. 127(85.8%) and 21(14.2%) patients had SMD >12 cm and SMD <12 cm, respectively. but the 6 difficult to intubate patients were from SMD >12 cm group. Out of 130(87.7%) Mallampati class I & II patients only 3(2.3%) case of difficult intubation was observed, but out of 18(12.16%) patients who exhibited Mallampati class III&IV, difficult intubation was encountered in 5(27.8%). Furthermore, from 18 patients who developed CL laryngoscope grade III&IV (difficult laryngoscopy), 15(83.3%) had Mallampati class of III and IV (p < 0.05) (Table 2).

**Table 2:** Preoperative airway parameters and their distribution with difficult laryngoscopy and intubation among surgical patients in Tikur Anbessa Specialized Hospital.

Predicators	Frequency, n (%)	DL, n (%)	DI, n (%)
Mallampati grade			
I and II	130(87.7%)	3(2.3%)	3(2.3%)
III and IV	18(12.16%)	15(83.3%)	5(27.8%)
TMD			
<6.5 cm	16(10.8%)	4(25%)	4(25%)
≥6.5 cm	132(89.2%)	14(10.7%)	5(3.8%)
SMD			
<12 cm	21(14.2%)	14(66.7%)	3(14.3%)
≥12 cm	127(85.8%)	4(3.1%)	6(4.7%)
IID			
≥3 cm	132(89.2)	15(11.36%)	7(5.3%)
<3 cm	16(10.8)	3(18.8%)	2(12.5%)
ULBT class Class I Class II and III	120(81.1%) 28(18.9%)	12(10%) 6(21.4%)	6(5%) 3(10.7%)
HRTMD ≤23 >23	117(79.1%) 31(20.9%)	4(3.4%) 14(45.2%)	6(5.1%) 3(9.7%)
			

### 3.3 Preoperative predictive values of difficult laryngoscopy and difficult intubation

#### 3.3.1 Predictive values for difficult laryngoscopy

In our study we have found that upper lip bite test (ULBT) had a higher sensitivity 90.2% and negative predictive value of 85.3%. Mallampati had a sensitivity of 45.8% and negative predictive value of 86%. IID also showed higher sensitivity (93.5%) and NPV of 87.5%. Sterno-mental distance showed higher specificity (91.1%) in predicting difficult laryngoscopy (Table 3). A multivariate analysis identified Mallampati class, Upper Lip Bite Test (ULBT) and IID as independent predictors for difficult laryngoscopy (p < 0.05)

**Table 3:** Sensitivity, specificity, positive predictive values, and negative predictive values for preoperative parameters against difficult laryngoscopy among surgical patients.

Test	Sensitivity %	Specificity %	PPV %	NPV%	P Value	Area	Accuracy	95% CL	
								lower upper	
Mallampati	45.8	65.9	20.4	86.2	0.004	0.96	96	0.882	0.981
TMD	58.3	22%	12.6	73	0.016	0.416	41.6	0.284	0.549
SMD	41.7	91.1	47.6	88.2	0.001	0.622	62.2	0.485	0.758
IID	93.5	50	50	87.5	0.001	0.602	60.2	0.504	0.780
HRTMD	68.6	70.8	54.8	73.2	0.01	0.659	65.9	0.527	0.722
ULBT	90.2	16.7	80	85.3	0.001	0.583	58.3	0.440	0.722

PPV = positive predictive value, NPV = negative predictive value.

#### 3.3.2 Predictive values for difficult intubation

Our study found that higher accuracy to predict difficult intubation in Mallampati and HRTMD (77.9%, and 80.3%, respectively). Mallampati grade, Thyromental distance and HRTMD have also showed greater sensitivity (69.6%, 58.3% and 47.8%, respectively) when compared to other tests. A higher specificity was seen in ULBT (80.2%) and SMD (79.6%). The negative predictive value of most of the pre-operative airway parameters were higher (Table 4). A multivariate analysis identified Mallampati class, Thyromental distance and HRTMD as independent predictors for difficult intubation (p < 0.001).

**Table 4:** Sensitivity, specificity, positive predictive values, and negative predictive values for preoperative parameters against difficult intubation.

Test	Sensitivity %	Specificity %	PPV	NPV	P – Value	Area	Accuracy %	95% CI	
								lower	Upper
Mallampati	69.6	65.6	30.6	92.6	0.002	0.779	77.9	0.644	0.914
TMD	58.3	22	12.6	73	0.014	0.456	45.6	0.255	0.650
SMD	34.8	79.6	38.1	88.2	0.017	0.661	66.1	0.453	0.87
IID	34	50	53.6	88.6	0.024	0.679	67.9	0.468	0.89
HRTMD	47.8	84	35	89.7	0.044	0.803	80.3	0.641	0.964
ULBT	17.4%	80.2%	80.4	86.7	0.001	0.60	60	0.386	0.816

PPV = positive predictive value, NPV = negative predictive value.

## 4 Discussion

This study found that magnitude of 12.2% and 6.1% for difficult laryngoscopy and intubation, respectively, among elective surgical patients with apparently normal airway. This result was in line with previous study from Ethiopia which showed the magnitude of difficult laryngoscopy and intubation as 13.6% and 5%, respectively. Furthermore the study revealed that 33.3% of patients with difficult laryngoscopy were found to be difficult for intubation [2]. Other studies also reported similar findings [7,17]. The magnitude of difficult laryngoscopy in our study appeared to be higher compared to the other literature [18]. The possible explanation for this result may be due to our study was conducted in teaching hospital that most of the intubations were performed by student anesthetists. Another observational study which assessed 350 consecutive patients (322 non-obstetric, 28 obstetric) showed that tracheal intubation was difficult among 17 (4.9%) patients, of whom four (1.14%) had a grade III or IV view on laryngoscopy. A Sternomental distance of 12.5 cm or less with the head fully extended on the neck and the mouth closed predicted 14 of the 17 patients in whom tracheal intubation was difficult [19].

In our study sensitivity, specificity, PPV and NPV Mallampati classification for difficult laryngoscopy was 45.8, 65.9%, 20.4% and 86.2%, respectively. Gupta et al. showed that the sensitivity, specificity, PPV and NPV of Mallampati class as 77.3%, 98.2%, 48.7% and 99.5%, respectively in predicting difficult airway (DAW). Harjai M and his colleagues from India also reported that among the clinical predictors, the Mallampati grading had the maximum receiver operating characteristic (ROC) and area under the curve (AUC) with 86.7% sensitivity to predict difficult laryngoscopy [7].

In accordance with our finding, a comparative study by George and Jacob in India on 141 surgical patients reported 54.5% sensitivity of Mallampati class as a predictor of difficult tracheal intubation [21]. Gupta AK. Et al., also reported similar findings [20].

ULBT was found to have sensitivity of 90.2% and specificity of 16.7% to predict difficult laryngoscopy. Comparable findings were also reported by other authors [22,23]. This study also found that SMD had sensitivity, specificity, PPV and NPV of 41.7%, 91.1%, 47.6% and 88.2%, respectively to predict difficult laryngoscopy. Dawit T et al. in Ethiopia conducted prospective observational study among 120 elective surgical patients and found that the sensitivity, specificity, PPV and NPV of 0%, 97.5%, 0% and 100% respectively [23]. A low PPV indicates test failure to answer the anesthetist's question regarding how likely would be the difficult intubation given that the test result was positive.

There is still no single test with 100% sensitivity and specificity to predict difficult laryngoscopy and intubation. The finding of our study also revealed that Mallampati classifications had sensitivity of 69.6% and specificity 65.6% for difficult intubation, and IID with sensitivity of 34% and specificity of 50% for predicting difficult laryngoscopy. This was comparable with a prospective observational study conducted by Hailekiros AG et al. in Gondar University hospital referral hospitals, Ethiopia which revealed that the sensitivity and specificity of Mallampati was 65% and 90%, and IID 73% and 81, respectively [4]. Similarly, another study also found the sensitivity, specificity, and the positive predictive values for the airway predictors as follows: Modified mallampati class (61.5%, 98.4%, 57.1%), TMD (15.4%, 98.1%, 22.2%), SMD (0%, 100%, 0%), and inter-incisor gap (30.8%, 97.3%, 28.6%) [17].

Available literatures did not show the predictive value of difficult laryngoscopy for difficult intubation. A good predictive test should have high sensitivity, specificity, positive and negative predictive values. Moreover, it should be simple enough to allow routine clinical use during preoperative evaluation and versatile enough so as to be applicable to different ethnic groups, gender and age. However high sensitivity is desirable as it will identify most patients in whom intubation will truly be difficult. In our study, HRTMD and Mallampati grade showed better accuracy (65.9% and 96%), respectively for difficult laryngoscopy. In addition, the accuracy of Mallampati class and HRTMD in predicting difficult intubation was 77.9% and 80.3%, respectively. According to Shiga et al. screening tests included were Mallampati classification, thyromental distance, sternomental distance, mouth opening, and Wilson risk score. Each test yielded poor to moderate sensitivity (20–62%) and moderate to fair specificity (82–97%). In contrast to the above findings, Srinivasa et al. and Savva showed greater sensitivity, specificity, and positive predictive values for most of the preoperative airway tests. This may be due to differences in patients’ physical appearance, sample size, and cutoff values for the screening tests [13,19,24].

In general our study found that Mallampati class, ULBT and IID as independent predictors for difficult laryngoscopy (p < 0.05), whereas Mallampati class, Thyromental distance and HRTMD as independent predictors for difficult intubation (p < 0.001). Similar finding was reported by a cross-sectional study from Gondar, Ethiopia which evaluated the magnitude and predisposing factors for difficult airway among 212 patients and showed Mallampati as a predictor of difficult airway [25].

## 5 Conclusion

Based on the result, we conclude that the magnitude of difficult intubation is not quite small that the anesthetists should expect difficult airway management in apparently normal patients.

Moreover, not all preoperative screening tests reliably indicate difficult intubation when used alone. Combination of some tests may have favorable effect in predicting true difficulty. However, Mallampati class, Thyromental distance and HRTMD can predict the probability of difficult endotracheal intubation in adult patients. Whereas Mallampati class and upper lip bite test (ULBT) predicts higher probability for difficult laryngoscopy.

## Limitation of the study

The limitation of this study includes it is a single centre study that it is only representative for the study hospital. Nevertheless, it is most likely that studies in other hospitals would lead to similar results. Furthermore, the study was focused on apparently normal individuals that we recommended large scale multicenter studies targeting the high risk populations.

## Ethical approval

Ethical approval obtained from Addis Ababa University department of anaesthesia and informed consent obtained from patients.

## Source of funding

This work was funded by 10.13039/501100007941Addis Ababa University. The study sponsors have no role in the collection, analysis and interpretation of data; in the writing of the manuscript; and in the decision to submit the manuscript for publication.

## Author contribution

All authors have made substantial contributions to conception, design, participated in the critical review, and editing of the manuscript drafts for scientific merit and depth.

## Declarations of competing interest

None.

## Research registration

It has been registered with the Research Registry with unique identifying number of researchregistry 6882.

## Guarantor

All authors.

## Availability of data and material

The data were collected by data collectors and submitted to the authors who are willing to share the data upon request from peer researchers.

## Provenance and peer review

Not commissioned, externally peer-reviewed.

## Abbreviations

ASA: American society of Anesthesiologists
BMI: Body Mass Index
C-L: Cormack –Lehane's
DTI: Difficult Tracheal Intubation
ETT: Endotracheal tube
HRTMD: Ratio of Height to Thyromental Distance
IID: Inter Incisor Distance
MMC: modified Mallampati classification
MO: Mouth opening
NPV: negative predictive value
OR: operation Room
PPV: positive predictive value
ROM: |Range of motion
SMD: Sternomental distance
SPSS: Statistical package for Social Science
TMD- T: hyromental distance
ULBT: Upper lip bite test
